# Infantile Myelofibrosis and Myeloproliferation with CDC42 Dysfunction

**DOI:** 10.1007/s10875-020-00778-7

**Published:** 2020-04-17

**Authors:** Jeffrey M. Verboon, Dilnar Mahmut, Ah Ram Kim, Mitsutoshi Nakamura, Nour J. Abdulhay, Satish K. Nandakumar, Namrata Gupta, Thomas E. Akie, Amy E. Geddis, Becky Manes, Meghan E. Kapp, Inga Hofmann, Stacey B. Gabriel, Daryl E. Klein, David A. Williams, Haydar A. Frangoul, Susan M. Parkhurst, Genevieve M. Crane, Alan B. Cantor, Vijay G. Sankaran

**Affiliations:** 1 Division of Hematology/Oncology, Boston Children’s Hospital and Department of Pediatric Oncology, Dana-Farber Cancer Institute, Harvard Medical School, Boston, MA USA; 2 Broad Institute of MIT and Harvard, Cambridge, MA USA; 3 Division of Basic Sciences, Fred Hutchinson Cancer Research Center, Seattle, WA USA; 4 Cancer and Blood Disorders Clinic, Seattle Children’s Hospital, Seattle, WA USA; 5 Department of Pediatrics, Vanderbilt University School of Medicine, Nashville, TN USA; 6 Department of Pathology, Microbiology, and Immunology, Vanderbilt University Medical Center, Nashville, TN USA; 7 Division of Hematology, Oncology, and Bone Marrow Transplantation, Department of Pediatrics, University of Wisconsin, Madison, WI USA; 8 Department of Pharmacology, Yale Cancer Biology Institute, Yale University School of Medicine, West Haven, CT USA; 9 The Children’s Hospital at TriStar Centennial, Sarah Cannon Research Institute, Nashville, TN USA; 10 Department of Pathology and Laboratory Medicine, Weill Cornell Medicine, New York, NY USA

**Keywords:** Primary Myelofibrosis, Rho GTPases, bone marrow microenvironment

## Abstract

Studies of genetic blood disorders have advanced our understanding of the intrinsic regulation of hematopoiesis. However, such genetic studies have only yielded limited insights into how interactions between hematopoietic cells and their microenvironment are regulated. Here, we describe two affected siblings with infantile myelofibrosis and myeloproliferation that share a common de novo mutation in the Rho GTPase CDC42 (Chr1:22417990:C>T, p.R186C) due to paternal germline mosaicism. Functional studies using human cells and flies demonstrate that this CDC42 mutant has altered activity and thereby disrupts interactions between hematopoietic progenitors and key tissue microenvironmental factors. These findings suggest that further investigation of this and other related disorders may provide insights into how hematopoietic cell-microenvironment interactions play a role in human health and can be disrupted in disease. In addition, we suggest that deregulation of CDC42 may underlie more common blood disorders, such as primary myelofibrosis.

## Introduction

Recent studies have provided considerable insights into how interactions between hematopoietic stem/progenitor cells and their microenvironment are regulated [5, 10, 34, 44]. Despite the substantial progress in this field, it remains unclear whether perturbation of such interactions can lead to human disease. Rare experiments of nature provide a potentially effective way to gain insights into how these interactions function normally in health and can be perturbed in disease states. For instance, a recent study demonstrated that chromothriptic disruption of a gain-of-function mutation in *CXCR4* in hematopoietic stem cells (HSCs) was curative in a patient with warts, hypogammaglobulinemia, immunodeficiency, and myelokathexis (WHIM) syndrome as a result of preferential expansion of the CXCR4-haploinsufficient HSCs in the bone marrow niche [28]. How other genetic blood disorders may alter hematopoietic cell-microenvironment interactions remains largely unknown.

In the context of acquired blood disorders, there is more evidence for altered interactions between hematopoietic cells and the bone marrow microenvironment. Mouse models have demonstrated that perturbations of the HSC niche can impact the function and differentiation of hematopoietic cells, as exemplified by mutations in the bone marrow microenvironment causing myeloproliferative disorders [34, 47, 48]. Moreover, studies of human primary myelofibrosis (PMF) have provided evidence for microenvironment dysfunction contributing to disease pathogenesis [23], and recent studies have elucidated pathways that may contribute to this process in mouse models [3, 12, 38, 40].

The Rho family of small GTPases composed of the RHO, RAC, and CDC42 proteins, which are best known as signaling molecules that control actin dynamics, have emerged as potent factors in hematopoiesis [31]. Indeed, this family of proteins is known to regulate hematopoietic cell shape, polarity, adhesion, and migration. Germline mutations of the hematopoietic-specific Rho GTPases *RAC2* and*RHOH* have previously been implicated in inherited immunological conditions, impacting myeloid and lymphoid cell functions [11, 50]. Recently, human mutations in *CDC42* have presented with diverse developmental phenotypes, and a specific missense mutation, R186C, has been reported in a few patients with a syndrome involving immune dysregulation [14, 22, 27].

Here, we have identified two children born to unaffected parents presenting with infantile myelofibrosis and myeloproliferation. Both affected patients harbor the identical CDC42 R186C mutation due to low-level paternal mosaicism. We demonstrate dysfunctional activity of this mutant form of CDC42. Introduction of this mutation into primary human hematopoietic stem/progenitor cells disrupts the ability of the cells to migrate towards the key hematopoietic chemokine, CXCL12/SDF-1 [10]. We additionally demonstrate that this mutant acts in a dominant manner to disrupt the ability of *Drosophila* hematopoietic cells to migrate appropriately. Our studies extend the phenotypic spectrum of disorders resulting from mutations of Rho GTPases and specifically implicate the CDC42 R186C mutation in altering interactions of hematopoietic cells with the microenvironment. Moreover, studies of acquired primary myelofibrosis suggest that disruption of CDC42 may occur in hematopoietic progenitor cells in this disease, connecting the observations made in these rare cases to the pathogenesis of other, more common, acquired malignant hematopoietic disorders.

## Methods

### Study Approval

All family members provided written informed consent to participate in this study. The institutional review boards of Boston Children’s Hospital and Massachusetts Institute of Technology approved the study protocols.

### Whole-Exome Sequencing and Related Genetic Analyses

The patients described in this paper are part of a rare blood disorder cohort that has been studied through the use of whole-exome sequencing (WES), as previously described [1, 19, 20, 45]. WES in these cases was performed using genomic DNA obtained from peripheral blood samples of the patients. The resultant variant call file (in hg19 coordinates) was annotated with VEP v89 [30], and rare variants (based on ExAC v0.3.1 and GnomAD r2.0.2) [24] (http://gnomad.broadinstitute.org/) were identified using a combination of the Genome Analysis Toolkit, Bcftools, and Gemini [25, 29, 33]. No rare (< 0.01% allele frequency in ExAC and GnomAD) loss-of-function or missense variants were identified in any known blood cell disorder genes. All mutations were confirmed from genomic DNA samples of the patients or family members by Sanger sequencing.

### Targeted Amplicon Sequencing

A 205-bp region containing the mutation was amplified from all family members and unrelated controls. The PCR amplicons were processed, and paired-end sequencing was performed using a MiSeq instrument (Illumina).

### Lentiviral Constructs

CDC42 wild type (WT) and R186C mutant cDNA were cloned into the HMD lentiviral vector with EcoRI and XhoI digestion and ligation. FLAG-tagged CDC42 WT and FLAG-tagged CDC42 R186C cDNA were also cloned into HMD lentivirus vector with EcoRI and XhoI digestion and ligation. Transduced cells were identified based on GFP expression driven by the IRES-GFP from the HMD vector.

### Cell Culture and Lentiviral Transduction

HEK293T and NIH-3T3 cell lines were obtained and maintained in Dulbelcco’s Modified Eagle Medium (DMEM; Gibco) supplemented with 10% of fetal bovine serum (FBS; Atlanta Biologicals) and 1% of penicillin/streptomycin (GIBCO). Human primary adult bone marrow-derived CD34^+^ hematopoietic stem and progenitor cells (HSPCs) were obtained from Fred Hutchinson Cancer Research Center and maintained in StemSpan II and 100X CC100 (Stem Cell Technologies) with recombinant thrombopoietin at 50 ng/mL (TPO; Peprotech).

CDC42 WT and R186C mutant lentiviral constructs were transfected into HEK293T with transfection reagent FuGene (Promega) and helper plasmids, as previously described [19, 26]. The primary HSPCs were infected with virus during day 1 of culture. Primary HSPCs were then sorted for GFP on day 3 and processed for assays. Multiple donors were used for downstream assays and produced similar results. NIH-3T3 cells were infected with lentivirus and 48 h later, used for further downstream applications.

### Fly Strains and Genetics

Flies were cultured and crossed on yeast-cornmeal-molasses-malt extract media and maintained at 25 °C. The following hemocyte-specific drivers were used: P{*Pxn-GAL4*}II, P{*UAS-GFP*}II and P{*Pxn-GAL4*}III, P{*UAS-GFP*}III [43].

### Generation of Cdc42(R186C) Fusion Constructs and Transgenics

A Cdc42(R186C) mutation was made by introducing the R186C point mutation into the wild type *Drosophila* Cdc42 cDNA by PCR. UAS-Cdc42(R186C) was made by cloning the resulting Cdc42(R186C) cDNA into the *Kpn*I-*Xba*I sites of the pUASp vector [37]. These constructs were used to make germline transformants, as previously described [18]. The resulting transgenic lines (P{*w+*; *UAS-Cdc42(R186C)*}) were mapped to a single chromosome and shown to have non-lethal insertions. mCh-Cdc42 flies have been previously described [2].

### Transwell Migration Assay

A transwell migration assay (Costar; Corning) was performed with the lentiviral-infected and GFP-positive sorted human CD34^+^ HSPCs. Briefly, 3X10^5^ CD34^+^ cells were suspended in StemSpan II media in the upper chamber of the transwell plate. The cells were allowed to migrate to the lower chamber in which there was CXCL12/SDF-1 at a concentration of 100 ng/mL in StemSpan II medium. Migration was performed over the course of 4 h at 37 °C. After incubation, number of cells that migrated to the lower chamber were quantified.

### Cell Cycle Assay

Infected and GFP-positive sorted CD34^+^ cells were also evaluated for cell cycle phase progression differences. These cells were suspended in StemSpan II media (Stem Cell Technologies) and stimulated with CXCL12/SDF1-a at 300 ng/mL (Peprotech) for 1 h at 37 °C. During this 1 h incubation, 10 μM of EdU-Alexa647 (ThermoFisher) was also added to the cell suspension. The cells were fixed, permeabilized, and stained with Click-iT™ EdU Alexa Flour 647™ Flow Cytometry kit (ThermoFisher) following the manufacturer’s protocols. Cells were also stained with propidium iodide (Biolegend) at 200 ng/mL during the final step of the protocol. Cell cycle distribution was acquired by using a BD LSR II flow cytometer (BD Biosciences), and the FlowJo software was used to determine the percentage of cells in different phases of cell cycle.

### Flow Cytometry

Surface staining of CXCR4/CD184 was measured in infected and GFP-positive sorted CD34^+^ cells by flow cytometry. Cells were recovered in CD34^+^ culture media after sort and washed with cold PBS twice. Cells were re-suspended in 1% BSA in PBS and stained with 5-μL CD184-APC from a stock of 200 μg/mL (306509; BD Biosciences) for 30 mins on ice. Cells were washed once with PBS after staining and re-suspended in 1% BSA in PBS with propidium iodide (Biolegend) at 200 ng/mL. CXCR4 surface staining FACS was acquired using an Accuri C6 flow cytometer (BD Biosciences), and mean fluorescence intensity of CXCR4 was measured by using the FlowJo software.

### Mammalian Cell Microscopy

3T3 cells were transfected with either FLAG-tagged WT CDC42 or CDC42 R186C and were plated on fibronectin-coated slides and stained with Rhodamine-Phalloidin following manufacturer protocols (BK005; Cytoskeleton Inc). Mouse, anti-Flag antibody was used for CDC42 visualization (166,355; Santa Cruz). Cells were mounted with DAPI-containing medium (0100–20; Southern Biochem). Confocal micrographs were imaged on a Zeiss LSM 700 using a Zeiss 40x/1.4 Plan-Apochromat objective.

### Cell Morphology Analyses

Infected 3T3 cells were washed with PBS, treated with 0.25% trypsin (GIBCO) for 4 min at 37 C, deactivated with 10% FBS supplemented medium, and re-plated onto fibronectin (2 μg/cm^2^)-coated plates (Sigma Aldrich) for 45 mins. Morphology differences were noted and captured by using light microscopy at different magnifications.

### Western Blotting

After 48 h of infection, approximately 1 million 3T3 cells were plated onto fibronectin-coated plates and incubated at 37 °C for 1 h. Post-incubation, cells were washed with PBS twice and then lysed with cold radioimmunoprecipitation assay buffer supplemented with protease and phosphatase inhibitor cocktails (Santa Cruz). Proteins were quantified with DC protein assay (BioRad), run using the Mini-Protean TGX gel system (BioRad), and transferred to polyvinylidene fluoride membrane. Signal was detected by ECL Amersham Hyperfilm (GE Healthcare). Western blotting was performed with primary antibodies anti-CDC42 at 1:500 dilution (610928; BD Biosciences), anti-PAK1/2/3 at 1:1000 dilution (2604; CST), anti-pPAK at 1:1000 dilution (2601s; CST), and anti-GAPDH at 1:10,000 dilution (clone 6C5; Santa Cruz). HRP-conjugated goat anti-mouse and anti-rabbit (BioRad) were used at 1:20,000 dilution as secondary antibodies.

### Fly Microscopy

For migration studies, fly embryos were collected on grape agar plates at either 25 °C for 1 h and then aged at 18 °C for 16–20 h or at 25 °C for 1 h and then aged 12–15 h at 25 °C. The two regimens yielded indistinguishable results. Embryos were then dechorionated, dried for 5 min, transferred onto strips of glue dried onto No. 1.5 coverslips, and covered with series 700 halocarbon oil (Halocarbon Products, River Edge, NJ). For migration studies, 30-μm stacks were taken once every 5 min with a 0.5-μm step size over the course of 1.5 h. Individual hemocytes were imaged from top to bottom with a 0.5–1-μm step size. All imaging was performed at room temperature (22 °C). For live imaging, the following microscope setup was used: Revolution WD systems (Andor Technology Ltd., Concord, MA, USA) mounted on a Leica DMi8 (Leica Microsystems Inc., Buffalo Grove, IL, USA) with a 20x/0.7 NA objective lens and controlled by using the MetaMorph software. Images and videos were acquired with 488 nm, using an Andor iXon Ultra 897 EMCCD cameras (Andor Technology Ltd., Concord, MA, USA). For individual hemocyte images, the following microscope setup was used: Zeiss LSM 780 spectral confocal microscope (Carl Zeiss Microscopy GmbH, Jena, Germany) fitted with Zeiss 40x/1.4 oil Plan-Apochromat objective at 4× zoom. FITC (Alexa 488) fluorescence was excited with the 488-nm line of an argon laser, and detection was between 498 and 560 nm. Pinhole was typically set to 1.0 Airy units. Confocal sections were acquired at 0.5–1.0-μm spacing. All images were analyzed with Fiji [39]. Measurements of percent cell body protrusions were done manually. Measurements of average hemocyte velocity over 30 min were done using the manual tracking plugin in Fiji. Five random hemocytes per embryo were measured.

### Microarray Analysis

Normalized expression sets for PMF and control samples were accessed using the R package GEOquery from the GEO dataset GSE53482. Differential expression was performed as previously described using the Bioconductor package Limma [36].

### Immunohistochemical Analysis of Primary Myelofibrosis

PMF (*n* = 10 independent samples) or non-diagnostic findings/normocellular (*n* = 7 independent samples) bone marrow specimens were identified in the pathology archives at Weill Cornell/New York Presbyterian Hospital. Bone marrow specimens underwent standard histologic processing and were stained with an antibody against CDC42 (PA1-092, ThermoFisher Scientific) following optimization of staining conditions. All measured samples were stained together to ensure comparable staining. Level of CDC42 staining in megakaryocytes was performed using the Halo Image analysis platform (Indica Labs). Between 3 and 14 megakaryocytes were quantified per individual sample to ensure consistency.

### Statistical Analyses

All pairwise comparisons were performed using the 2-tailed Student’s *t* test, unless otherwise indicated. Differences were considered significant if the *p* value was less than 0.05. Statistical testing for deep sequencing was done using a one-sided binomial test, Bin(q,n,p), where*q* is the number of alternate alleles,*n* is the depth at the variant position, and *p* is the site-specific error rate determined from the unrelated samples.

### Data Availability

The whole-exome sequencing data are available in the dbGaP database (http://www.ncbi.nlm.nih.gov/gap) under the accession number phs000474.v2.p1.

## Results

### Case History

**Fig. 1:**
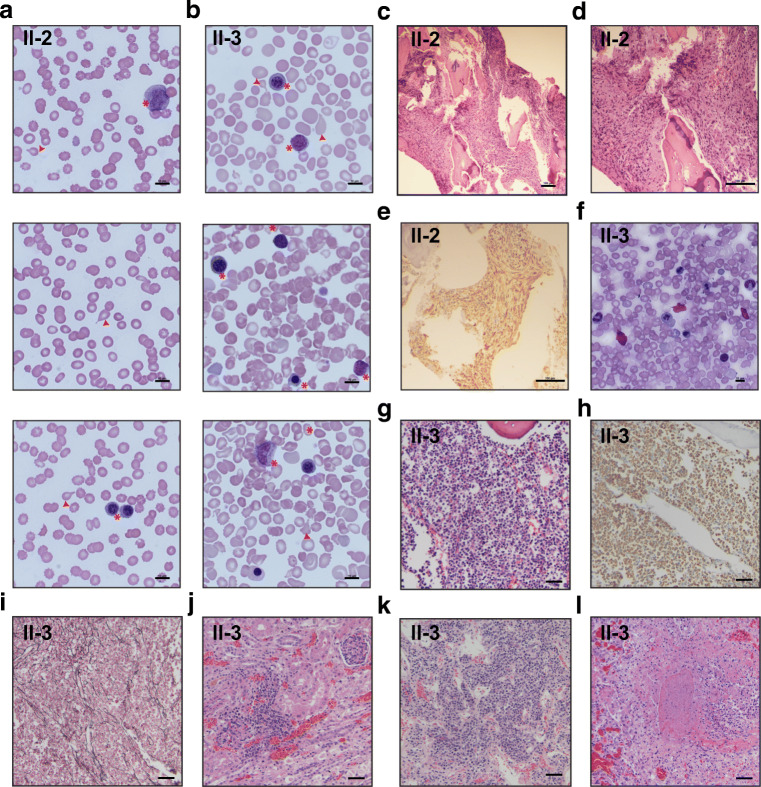
Hematologic profile of patients with infantile myelofibrosis. **a**, **b** Blood smears from patients II-2 and III-3 showing tear drops and leucoerythroblastic picture in the circulation. Shown at × 1000 magnification, scale bar = 10 μm. Teardrop cells are indicated by red arrows. Early myeloid and erythroid precursor cells are indicated with asterisks. **c** Hematoxylin and eosin-stained bone marrow section showing myelofibrosis from patient II-2. Shown at × 100 magnification, scale bar = 100 μm. **d** Hematoxylin and eosin (H&E)-stained bone marrow section showing myelofibrosis from patient II-2. Shown at × 200 magnification, scale bar = 100 μm. **e** Vimentin immunohistochemical-stained bone marrow showing myelofibrosis from patient II-2. Shown at × 200 magnification, scale bar = 100 μm. **f** Bone marrow aspirate from patient III-3 obtained on day of life 1 demonstrating erythroid dysplasia consisting of erythroid precursors with karyorrhexis, nuclear blebbing, and atypical nuclear contours. There is also left-shifted granulopoiesis. Shown at × 600 magnification, scale bar = 10 μm.**g–l** Autopsy findings of patient III-3, all shown at × 200 magnification, scale bars = 50 μm.**g** Representative sections of vertebral column demonstrate hypercellular bone marrow comprised exclusively of early myeloid cells (H&E) highlighted by **h** myeloperoxidase (MPO) immunostain with **i** mild fibrosis (reticulin stain). Representative sections of the **j** bilateral kidneys show multifocal interstitial early myeloid progenitor cells without evidence of extramedullary trilineage hematopoiesis (H&E). **k** Representative sections of lymph nodes show multifocal clusters of early myeloid progenitor cells predominantly within the medulla (H&E). **l** Lungs show multifocal necrotizing lesions with frequent neutrophils (H&E)

A description of the clinical course of the proband has previously been reported [41]. Briefly, he is a Caucasian male born at 37 weeks of estimated gestational age following labor induction due to maternal hypertension. No dysmorphic features were noted. On day of life 1, he developed a patchy and scaly erythematous rash that intermittently disappeared and recurred, and was urticarial at times. At 1 week of age during evaluation for his rash, he was found to be thrombocytopenic with a platelet count of 36 K/μL. He then developed fever and lethargy and was admitted to the hospital where he underwent a sepsis rule out, skin biopsy, and bone marrow biopsy. The skin biopsy revealed perivascular and interstitial inflammation with eosinophils. The bone marrow biopsy showed a hypercellular marrow with predominantly maturing myeloid elements, a high myeloid:erythroid ratio, and presence of megakaryocytes. Blasts were < 2% and the karyotype was normal 46 (X,Y). No hemophagocytosis was noted. He was discharged home, but about 1 week later, he was re-admitted after developing hematochezia associated with a platelet count of 10 K/μL. Hepatomegaly was noted for the first time, which progressively worsened. He also developed persistent tachypnea and intermittent hypoxia requiring supplemental oxygen. A chest X-ray was unremarkable. It was thought that his tachypnea was due to mechanical causes related to his marked hepatomegaly. Liver biopsy revealed extensive extramedullary hematopoiesis. He subsequently developed splenomegaly. He continued to have a recurrent polymorphic rash as well as intermittent angioedema and nodules on his hands. A ferritin level at 18 days of life was 594 ng/mL, and fibrinogen was 211 mg/dL. Repeat skin biopsy showed inflammatory infiltrates with lymphocytes, histiocytes, and rare neutrophils and eosinophils. He developed anemia and neutropenia in addition to his thrombocytopenia and became both red blood cell and platelet transfusion dependent. He also developed mild eosinophilia with absolute eosinophil counts ranging from 0.52 to 3.2 K/μL. Immunoglobulin levels were all elevated (IgM 202 mg/dL, IgG 1830 mg/dL, IgE 353 IU/mL, and IgA 81 mg/dL) except for IgD. Peripheral lymphocyte subset studies were normal. Repeated bone marrow biopsies revealed a hypercellular bone marrow with megakaryocyte dysplasia and moderate to marked fibrosis that was positive by reticulin and trichrome stains (Fig. 1). The karyotype continued to be normal, and no excess of blasts was found. In vitro GM-CSF hypersensitivity testing to assess for possible juvenile myelomonocytic leukemia was negative. He was diagnosed with idiopathic infantile myelofibrosis. He was treated with a 4-day course of cytosine arabinoside (Ara-C) at 20 mg/m^2^/day to try to reduce his hepatosplenomegaly, but he did not have a clinical response. He continued to have baseline hypoxia with massive hepatomegaly causing restrictive lung disease. Oxygen saturations were 88% breathing room air, but increased to > 94% with supplemental oxygen. Echocardiography did not show evidence of pulmonary hypertension, and chest CT did not show evidence of parenchymal disease. At 2 months of age, he underwent bone marrow transplantation (BMT) with his 4-year-old female HLA-matched sibling serving as the donor. He was conditioned for BMT with busulfan (1.2 mg/kg/dose every 6 h for 16 doses, with busulfan pharmacokinetics to achieve a range of 600 to 900 nm/mL) followed by cyclophosphamide, 50 mg/kg for 4 days. There was no radiation as part of the formal BMT conditioning regimen. However, he did receive 300 cGy to his spleen prior to BMT conditioning to try to reduce the spleen size because of mechanical interference with his respiration. The patient’s respiratory compromise improved in the first few days following BMT, and he no longer required supplemental oxygen. However, beginning on day 21 following BMT, he developed worsening tachypnea and hypoxia. There were no infiltrates on his chest X-ray. Cardiac catheterization demonstrated severe pulmonary hypertension with baseline right pulmonary arterial pressure of 70 mmHg compared with a systemic pressure of 65 mmHg. He was placed on diuretics and calcium channel blockers, but his respiratory disease worsened and he died on day 32 following BMT. Autopsy revealed severe intimal hyperplasia of the pulmonary arterioles.

**Fig. 2:**
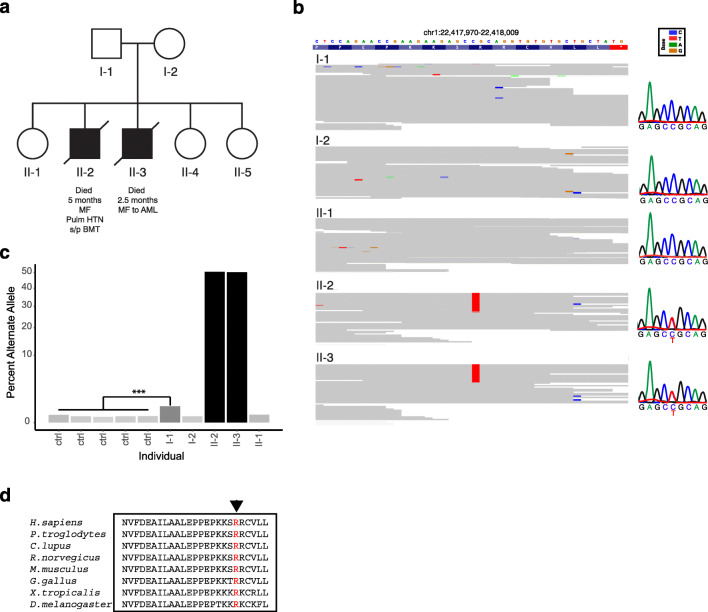
Identification of the R186C missense mutation in CDC42 in two patients with infantile myelofibrosis. **a** Pedigree of the kindred affected by infantile myelofibrosis. **b** Visualization of the variant (Chr1:22417990:C>T)-containing region showing the heterozygous variant in the two affected children, which is absent from all unaffected family members. This visualization was produced using the Integrated Genomics Viewer. Exome variant results were validated by using Sanger sequencing, as shown on the right. **c** Quantification of reads containing the Chr1:22417990:C>T (hg19 coordinates) variant from deep-sequenced amplicons. *P* values indicated from binomial test. **d** Alignment of CDC42 from diverse eukaryotes shows that the R186 residue is highly conserved

The proband’s younger brother (Fig. 2a, II-3) was born at 35 2/7 weeks of gestation by cesarean section due to antenatally detected hepatomegaly and reduced fetal movements. He was blue and floppy at birth (SaO2 in the 50% range with poor chest movement) and was intubated. Apgar scores were 3 at 1 min, 6 at 5 min, and 9 at 10 min. He was also noted to have a purpuric rash, hepatosplenomegaly, anemia (initial hematocrit 34%), reticulocytosis (initial reticulocyte count 9.8%), thrombocytopenia (initial platelet count 11 K/μL), leukoerythroblastosis (Fig. 1), and an elevated C-reactive protein level (73.5 mg/dL). A bone marrow aspirate performed on day of life 1 demonstrated moderate erythrocytic dysplasia. The karyotype was normal showing 46(X,Y). No hemophagocytosis was observed. Flow cytometric analysis revealed < 3% myeloblasts. A bone marrow core biopsy was not successful. He was weaned to continuous positive airway pressure (CPAP) by day of life 4, but continued to require supplemental oxygen. There was no evidence of pulmonary hypertension by echocardiography. It was thought that his severe hepatomegaly was causing restrictive lung disease. He was given supportive care while awaiting bone marrow transplantation. He remained platelet and red blood cell transfusion dependent, and continued to have elevated C-reactive protein levels (38.7–96.7 mg/dL). At about 4 weeks of life, he developed labored breathing and tachycardia, followed by bradycardia and decreased respiratory rate. He was re-intubated and diagnosed with presumed septic shock. Chest X-rays showed total atelectasis of the left lower lobe with bronchiectasis with the lobe. There was marked hyperexpansion of the left upper lobe and heavy markings of the right lung. Chest CT was initially concerning for possible congenital lobar emphysema, but this was subsequently ruled out. Direct laryngoscopy and bronchoscopy showed bronchomalacia below the right mainstem bronchus. He subsequently developed multi-organ failure and died at about 2.5 months of age. Autopsy revealed a hypercellular vertebral bone marrow comprised exclusively of early myeloid cells (Fig. 1g), which are positive for myeloperoxidase (MPO) via immunohistochemistry (Fig. 1h) and negative for CD3, CD43, CD34, and CD117. Mild fibrosis could be highlighted by reticulin stain (Fig. 1i), and patchy involvement was noted of the spleen, bilateral kidneys (Fig. 1j), and lymph nodes (Fig. 1k). There was diffuse trichome-positive fibrosis of the liver. The lungs were bilaterally consolidated with left upper lobe hemorrhage and necrotizing pneumonia (Fig. 1l) present in the left upper, left lower, and right lower lobes. There was no evidence of pulmonary hypertension. No fibrosis was noted. Lung cultures showed moderate growth of *Pseudomonas aeruginosa*.

The proband’s 3 sisters (II-1, II-4, II-5) and both parents (I-1, I-2) are all healthy. Sanger sequencing of peripheral blood mononuclear DNA from the proband and his affected younger brother failed to identify mutations in the coding sequences or intron/exon boundaries of *GATA1*, *JAK2*, or*MPL*.

### Genetic Analyses

Given the observed inheritance pattern, we initially searched for X-linked or autosomal recessive causes of this phenotype using WES data generated from all family members (Fig. 2a). This analysis was unrevealing for any putative causal mutations showing appropriate segregation. However, analysis of potential de novo variants revealed a single recurrent event in both affected children, Chr1:22417990:C>T (hg19 coordinates), resulting in the R186C mutation in*CDC42* (Fig. 2b). Given that such recurrent de novo mutations can be associated with low-level parental germline mosaicism [35] and that the father had shown a single read with this variant in WES, we performed deep sequencing to > 100,000-fold of the mutated region of *CDC42* in blood-derived genomic DNA from all family members and unrelated controls and observed an increased level of mosaicism from 0.07 to 0.15% in controls to 0.59% in the unaffected father (I-1; Fig. 2c). This finding suggests paternal germline mosaicism as the source for the recurrent mutations noted in the two affected children. This mutation was located in an evolutionarily conserved C-terminal polybasic region of CDC42 (Fig. 2d) and was recently described in patients with a distinct disorder characterized by immune dysregulation [14, 22].

### Functional Studies

CDC42 is a member of the well-studied Rho family of small GTPases and, through interacting proteins known as effectors, regulates signaling pathways that control diverse cellular functions including cell cycle progression, cell migration, and cytoskeletal dynamics [13]. Rho GTPases have been shown to play critical roles in hematopoietic cells, including hematopoietic stem/progenitor cells, as well as cells of the myeloid and lymphoid lineages [31]. In addition, Rho GTPases, particularly RHOA, RHOH, and the GTPase most closely related to CDC42, RAC, have been implicated in malignant transformation. CDC42 is best studied for its role in regulating and enabling signaling pathways to intersect with cytoskeletal activity. Importantly, CDC42 has been shown to have a critical role in HSCs and other hematopoietic progenitors through the study of mutant mice [52, 53]. Conditional deletion of *Cdc42* in the mouse hematopoietic system led to a rapidly fatal myeloproliferative disorder [53]. Finally, a different variant in *CDC42* (Y64C) in a human patient has been associated with myelofibrosis observed in adulthood [7]. Therefore, the observed mutation appeared to be potentially relevant to hematopoiesis, although the human phenotype described in this kindred is distinct from what has been reported in animal models and other human patients previously.

**Fig. 3:**
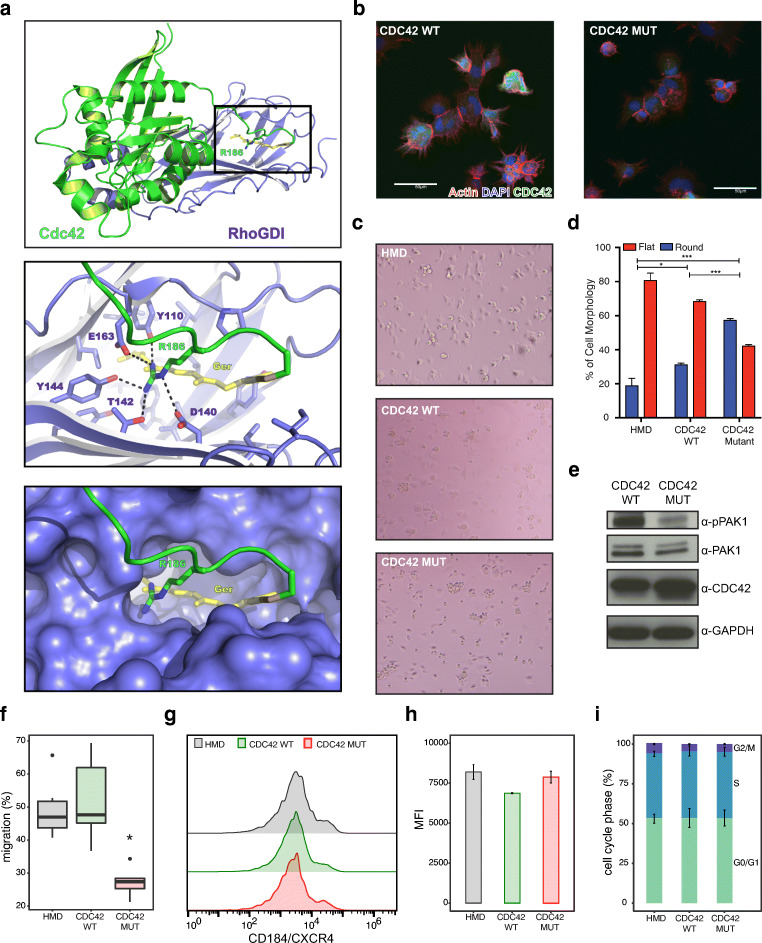
Deregulated activity of the CDC42 R186C mutation leads to altered cell migration. **a** A visualization using Pymol of the interaction between CDC42 and RhoGDI with the critical interactions by the R186 residue highlighted in the zoomed in bottom two panels. **b** Confocal micrographs of 3T3 cells infected with FLAG-tagged WT CDC42 showing normal filopodia formation when plated on fibronectin-coated slides. In contrast, the FLAG-tagged CDC42 R186C mutant cells lack filopodia. Scale bars 50 μm. **c** Light microscopic images of 3T3 cells infected with an empty HMD vector, WT CDC42, or mutant CDC42 plated onto fibronectin-coated plates for 45 min. HMD and WT CDC42-infected cells showed a higher percentage of flat cells, as compared with the presence of rounded cells. **d** Quantification (mean ± SEM) of observed flattened versus rounded 3T3 cell morphology (the total number of cells assessed per condition was HMD = 637, WT CDC42 = 462, and CDC42 R186C= 545, which were quantified across 3 biological replicates). **e** Protein lysates of 3T3 cells infected with WT CDC42 and mutant CDC42 vectors analyzed by western blotting. PAK1 expression remains the same between two conditions, but phosphorylated PAK1 shows reduced expression. Representative experiment is shown.**f** CD34^+^ HSPCs infected with the control vector or WT CDC42 show more migration towards CXCL12/SDF-1 over a 4 h time period in a transwell migration assay, compared with cells overexpressing mutant CDC42 R186C (mean ± SD, *n* = 6, *p < 0.05, two-tailed *t* test). **g** CXCR4 surface expression of infected CD34^+^ HSPCs with respective vectors shows similar expression patterns. **h** Quantification of CD184/CXCR4 mean fluorescence intensity of different conditions shows little variation (mean ± SD, *n* = 3, two-tailed *t* test). **i** The percentage of CD34^+^ HSPCs in various cell cycle phases show no difference among HMD-, WT CDC42-, and mutant CDC42-transduced cells (mean ± SD, *n* = 6, two-tailed*t* test)

We initially examined the location of the mutation in CDC42 in a structure involving the complex of CDC42 with its critical guanine nucleotide exchange factor, RhoGDI (encoded by the *ARHGDIA* gene in humans; Fig. 3a) [17]. The R186 residue is located near the geranylgeranyl moiety and is predicted to alter several key interactions with RhoGDI (Fig. 3a), as has recently been demonstrated through functional analyses [22]. Consistent with this, introduction of either wild type or the R186C mutant CDC42 into 3T3 cells using lentiviral transduction led to abnormal cytoskeletal structure and cell morphology. When transduced cells were plated on fibronectin, we found that the mutant, but not control or WT, CDC42-transduced cells inhibited the formation of filipodia, which depend upon CDC42 activity (Fig. 3b) [16]. After short-term plating of 3T3 cells onto fibronectin for 45 min, we noted that significantly more control or WT CDC42-transduced cells showed a flattened morphology compared with the CDC42 mutant-transduced cells that demonstrated a predominantly rounded morphology (Fig. 3 c, d). These results suggested that the mutant was acting in a dominant-negative or potentially neomorphic manner. Additionally, the phosphorylation of PAK1, a known CDC42 effector protein, was reduced in the mutant-transduced 3T3 cells plated on fibronectin compared with WT CDC42-transduced cells (Fig. 3e).

While these studies suggested dominant-negative or neomorphic effects and deregulated activity of the mutant CDC42, we wanted to gain more precise insights into the function of this mutant protein in hematopoietic cells. Since no hematopoietic cells were available from the patients, we utilized CD34^+^ HSPCs from healthy individuals. These cells were transduced using lentiviral vectors expressing either the WT or the R186C mutant form of CDC42. Two days after infection and immediately after flow cytometric sorting of the transduced cells, we assessed cell migration in a CXCL12/SDF-1 gradient using a transwell migration assay (Fig. 3f). While we observed no change in the expression of CXCR4 (CD184), the receptor for CXCL12/SDF-1 (Fig. 3 g, h), we noted a > 2-fold reduction in chemokine-directed migration of cells (Fig. 3f). We next assessed cell cycle progression in vitro using BrdU staining in the HSPCs transduced with either the control, WT, or mutant CDC42 in the presence of multiple cytokines including thrombopoietin, stem cell factor, interleukin 3 (IL-3), and the chemokine, CXCL12. Under these conditions, we noted no difference in cell cycle progression (Fig. 3i), suggesting that the observed phenotypes in the patients rely upon disruption of key interactions in the context of an intact niche, as has been observed in conditional knockout mice [53].

As this CDC42 residue is highly conserved (Fig. 2d), and hematopoiesis in *Drosophila melanogaster* has many shared features with mammalian hematopoiesis, including critical interactions between hematopoietic cells and their microenvironment; we further explored this mutation in vivo in *Drosophila* [4]. Rho GTPases have previously described important functions in the fly hematopoietic system, where Rho1 and its downstream effector Wash have key roles in the migration of embryonic hemocytes, while Cdc42 has been shown to be critical for maintaining appropriate cell polarity during hemocyte migration towards wounds [43, 46].

**Fig. 4:**
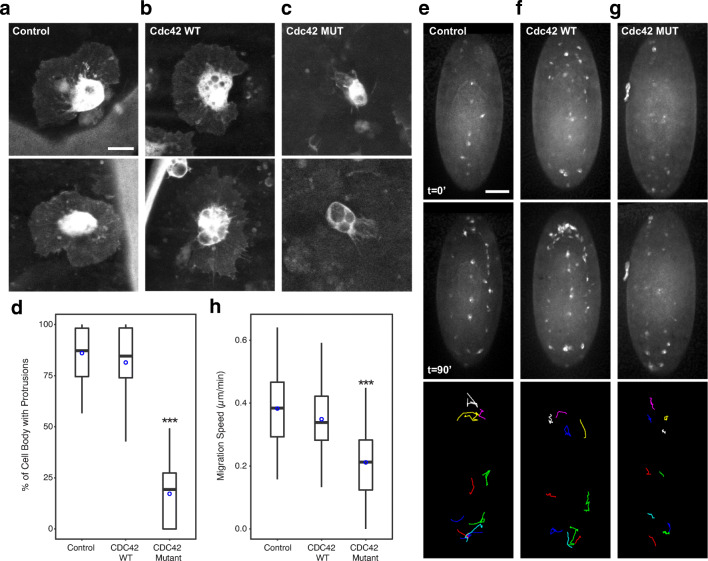
Defective migration due to dominant-negative or neomorphic Cdc42 activity in *Drosophila* hemocytes. **a**–**c** Confocal projections of hemocytes in GFP-expressing embryos of control (Gal4 driver alone) (**a**), UAS-Cdc42 WT (**b**), and UAS-Cdc42 mutant (**c**). **d** Boxplot of the percentage of the hemocyte cell body with protrusions (*n* ≥ 20). **e–g** Time-lapse series of ventral surface projections of control (**e**), UAS-Cdc42 WT (**f**), and UAS-Cdc42 mutant (**g**) migrating hemocytes expressing GFP. T = 0 min (top panel) and t = 90 min (middle panel) time points are shown. Random hemocytes were tracked every 5 min for 90′ (bottom panel) and show reduced migration distances.**h** Boxplot of hemocyte migration speed (*n* = 100). *P* values are indicated. Mean values are indicated by blue circles. Scale bars 10 μm for**a**–**c** and 50 μm for**e**–**g**

In order to explore this specific mutation in *Drosophila*, we made transgenic flies which express the CDC42 R186C mutation using the inducible UAS-Gal4 system in Pxn-Gal4/UAS-GFP transgenic flies to express this mutant CDC42 specifically in *Drosophila* hemocytes that also expressed GFP [6, 37]. We explored hemocyte function in wild type flies containing the Gal4 driver only (control), flies overexpressing wild type Cdc42 (Cdc42 WT), and flies overexpressing the Cdc42 R186C mutation (Cdc42 mutant) and found that in contrast to control and Cdc42 WT flies, Cdc42 mutant flies had reduced protrusions in migrating hemocytes (Fig. 4a–c). In order to quantify this defect, we measured the percentage of the hemocyte cell body with protrusions and found a profound loss of protrusions in the Cdc42 mutant compared with control and Cdc42 WT hemocytes (*p* < 0.0001) (Fig. 4d). Given the defective migration observed with the CDC42 mutant in human hematopoietic cells, we wondered whether *Drosophila* hemocytes may also fail to migrate appropriately from head to tail in response to cues supplied by their microenvironment. During fly embryogenesis, hemocytes are formed from the head mesoderm, while the head and tail are adjacent. A subset of hemocytes migrate to the tail from the head, dependent on the Rho GTPase, RhoL, before the tail retracts during morphogenesis to the posterior [42]. Once tail retraction is complete and in a chemotactic response to the PVR ligands, Pvf2 and Pvf3, head hemocytes migrate towards the posterior and tail hemocytes migrate anteriorly along the ventral midline towards each other and subsequently distribute throughout the entire embryo [9, 51]. This stereotypic process starts in stage 10 embryos and completes in ~ 2 h in normal embryos. We found that this migration process was severely disrupted in embryos expressing the Cdc42 mutant, but not in control or Cdc42 WT embryos (Fig. 4e–g,*n* = 20 replicates per condition). Despite this defect, hemocytes in all conditions were able to receive cues to migrate from head to tail and vice versa as hemocytes migrate from both the head and tail and can be seen along the ventral midline (Fig. 4g, 90 min). However, their capacity for migration was greatly diminished as Cdc42 mutant-expressing hemocytes showed ~ 2-fold reduction of migration speed, as compared with the other conditions (*p* < 0.0001, *n* = 5 hemocytes per embryo, 20 embryos per condition). Thus, the CDC42 R186C mutation in flies also functions in a dominant-negative or neomorphic manner, impacts cell protrusions, and impairs the ability of hematopoietic cells to migrate in response to tissue microenvironmental cues, leading to an overall defective hematopoietic system.

### Relationship to Primary Myelofibrosis

**Fig. 5:**
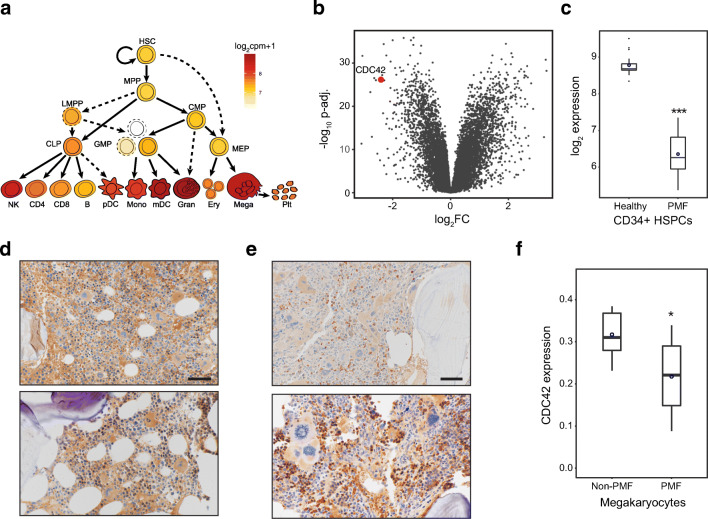
Potential role for CDC42 deregulation in primary human myelofibrosis. **a** Schematic of hematopoiesis showing log_2_ (cpm+1) expression from RNA sequencing of *CDC42* across the hematopoietic hierarchy.**b** A volcano plot showing log_2_ fold change (FC) of differentially expressed genes between CD34^+^ HSPCs from peripheral blood in control (*n* = 16) compared with PMF patient samples (*n* = 42) at indicated *P* values. *CDC42* is among the most downregulated genes in PMF CD34^+^ HSPCs. **c** Box plot depicting log_2_ expression of *CDC42* in CD34^+^ HSPCs from peripheral blood in control versus PMF patient samples shows a > 5-fold reduction in expression. **d**–**s** Representative immunohistochemical stains for CDC42 in the bone marrow of normocellular individuals (**d**) or individuals with PMF (**e**). **f** Quantification of CDC42 staining intensity in megakaryocytes from 10 PMF patients and 7 normocellular marrows for comparison. Between 3 and 14 megakaryocytes were measured from each individual. Mean values are indicated by blue circles

Primary myelofibrosis is a condition associated with somatic mutations in JAK2 or other factors that activate the thrombopoietin signaling pathway [15, 49]. Given the previously characterized role of Rho GTPases in hematopoietic cell localization within the marrow microenvironment [8, 31] and the disruption of this process observed in PMF [23], we hypothesized that the severe infantile myelofibrosis phenotype that results from the CDC42 R186C mutation may provide insights into the more common acquired PMF cases. CDC42 was broadly expressed in normal human hematopoiesis across the full hematopoietic hierarchy, including in HSCs and other early hematopoietic progenitors (Fig. 5a). To examine whether *CDC42* mRNA expression may be altered in PMF patients, we compared gene expression in peripheral blood-derived CD34^+^ HSPCs from 42 PMF cases with 16 healthy donors [32]. *CDC42* was among the most downregulated genes in PMF compared with normal donors with expression being ~ 5-fold less across all the samples (Fig. 5b, c). To validate these observations, we assessed bone marrow sections from 10 individuals with PMF and 7 individuals with normocellular bone marrows. We opted to examine megakaryocytes, given that they are easily distinguished morphologically and highly express CDC42 (Fig. 5a). We observed reduced CDC42 protein expression using immunohistochemistry in the megakaryocytes across the PMF patients, bolstering and extending our results on CDC42 dysfunction occurring more commonly in PMF (Fig. 5d, e, f).

## Discussion

Our findings complement recent studies of patients with a distinct disorder involving immune deregulation [14, 22] and suggest that CDC42 dysfunction can result in congenital myelofibrosis and myeloproliferation presenting during infancy. The data reported here extend the phenotypic spectrum resulting from CDC42 R186C mutations and suggest that patients presenting with congenital myeloproliferation or myelofibrosis be screened for this germline mutation. The differences between the phenotypes reported here and those reported recently with similar *CDC42* mutations are of interest and warrant further investigation [14, 27]. We would note that there is clear phenotypic overlap between the patients reported here and the other patients previously reported to have a syndrome of immune dysregulation due to the R186C mutation [22]. Some of the phenotypes reported in the other patients, such as the observed alterations in hematopoiesis, may be attributable to altered interactions between hematopoietic progenitors and the microenvironment, as we demonstrate here. In addition, immune dysregulation may also be contributing to the observed phenotypes and perturbation of hematopoiesis. Further studies to dissect these varying contributions will be valuable.

Additional study of these disorders might provide insights into the role of CDC42 in the intrinsic regulation of hematopoietic cells in their microenvironment, as we suggest through our functional studies of human and fly hematopoietic cells. It is notable that both the upstream receptor, CXCR4, and the downstream effector, WASP, which act in concert with CDC42, have key roles in enabling HSCs to home to and occupy their appropriate niche in the bone marrow [21, 28]. Through rare experiments of nature, we demonstrate how disruption of these key hematopoietic cell-microenvironment interactions may result in human disease and may also have implications for more common acquired blood disorders, such as PMF.
